# Two Distinct Lysosomal Targeting Strategies Afford Trojan Horse Antibodies With Pan-Filovirus Activity

**DOI:** 10.3389/fimmu.2021.729851

**Published:** 2021-10-14

**Authors:** Ariel S. Wirchnianski, Anna Z. Wec, Elisabeth K. Nyakatura, Andrew S. Herbert, Megan M. Slough, Ana I. Kuehne, Eva Mittler, Rohit K. Jangra, Jonathan Teruya, John M. Dye, Jonathan R. Lai, Kartik Chandran

**Affiliations:** ^1^ Department of Microbiology and Immunology, Albert Einstein College of Medicine, Bronx, NY, United States; ^2^ Department of Biochemistry, Albert Einstein College of Medicine, Bronx, NY, United States; ^3^ Virology Division, United States Army Medical Research Institute of Infectious Diseases, Frederick, MD, United States; ^4^ The Geneva Foundation, Tacoma, WA, United States; ^5^ Antibody Discovery and Research group, Mapp Biopharmaceutical, San Diego, CA, United States

**Keywords:** filovirus, NPC2, IGF2, Ebola, Marburg, Trojan Horse bispecific antibodies, NPC1, cryptic epitopes

## Abstract

Multiple agents in the family *Filoviridae* (filoviruses) are associated with sporadic human outbreaks of highly lethal disease, while others, including several recently identified agents, possess strong zoonotic potential. Although viral glycoprotein (GP)-specific monoclonal antibodies have demonstrated therapeutic utility against filovirus disease, currently FDA-approved molecules lack antiviral breadth. The development of broadly neutralizing antibodies has been challenged by the high sequence divergence among filovirus GPs and the complex GP proteolytic cleavage cascade that accompanies filovirus entry. Despite this variability in the antigenic surface of GP, all filoviruses share a site of vulnerability—the binding site for the universal filovirus entry receptor, Niemann-Pick C1 (NPC1). Unfortunately, this site is shielded in extracellular GP and only uncovered by proteolytic cleavage by host proteases in late endosomes and lysosomes, which are generally inaccessible to antibodies. To overcome this obstacle, we previously developed a ‘Trojan horse’ therapeutic approach in which engineered bispecific antibodies (bsAbs) coopt viral particles to deliver GP:NPC1 interaction-blocking antibodies to their endo/lysosomal sites of action. This approach afforded broad protection against members of the genus *Ebolavirus* but could not neutralize more divergent filoviruses. Here, we describe next-generation Trojan horse bsAbs that target the endo/lysosomal GP:NPC1 interface with pan-filovirus breadth by exploiting the conserved and widely expressed host cation-independent mannose-6-phosphate receptor for intracellular delivery. Our work highlights a new avenue for the development of single therapeutics protecting against all known and newly emerging filoviruses.

## Introduction

Several members of the family *Filoviridae* of enveloped viruses (filoviruses), including Ebola virus (EBOV), Bundibugyo virus (BDBV), Sudan virus (SUDV) and Marburg virus (MARV), cause outbreaks of highly lethal disease in humans (1). Moreover, multiple novel filoviruses with unknown potential for human emergence, including Lloviu virus (LLOV), Bombali virus (BOMV), and Měnglà virus (MLAV), have been discovered in the past decade (2–6). Although monoclonal antibody (mAb) therapeutics, such as ZMapp™, REGN-EB3 (Inmazeb™), and mAb114 (Ebanga™), have shown promise in human outbreaks, with the latter two having received FDA approval, they lack antiviral breadth (7–10). Specifically, these EBOV therapeutics cannot recognize and block infection by any other filovirus (7, 8, 11–13). Given the logistical and practical challenges inherent in developing filovirus-specific therapeutics, recent attention has turned to the identification of broadly neutralizing mAbs and cocktails, including MBP134, a two-mAb pan-ebolavirus cocktail that could protect nonhuman primates against challenge with EBOV, BDBV, and SUDV (14, 15). However, no mAb-based therapeutics with true pan-filovirus breadth have been identified to date, concordant with the limited conservation in the antigenic surface of the viral entry glycoprotein (GP) across filovirus species and differences in viral epitope shielding due to species-specific variations in GP glycosylation (16).

The receptor-binding site (RBS) is one GP epitope that is conserved across filoviruses. However, this potential viral ‘Achilles heel’ is shielded by the glycan cap subdomain in ebolavirus GPs and exposed only upon GP proteolytic processing by host cysteine cathepsins in cellular endo/lysosomal compartments (16–19). This critical step in viral entry occurs after viral internalization and endocytic trafficking and generates a cleaved form of GP (GP_CL_) that is competent to engage the essential and universal filovirus entry receptor, Niemann-Pick C1 (NPC1) (19–26).

Unfortunately, the cryptic nature of the RBS epitope and the intracellular location of GP_CL_:NPC1 association renders this conserved virus-receptor interaction largely inaccessible to conventional antibodies. To overcome this obstacle, we previously developed a ‘Trojan horse’ bispecific antibody (bsAb) strategy, in which RBS- and NPC1-targeted mAbs MR72 and mAb-548, respectively, were equipped with combining sites from a mAb, FVM09, that recognizes a conserved but non-neutralizing epitope in extracellular GP. These bsAbs were thus able to “hitch a ride” into the cell with infecting virions through FVM09 and successfully target the endo/lysosomal GP_CL_:NPC1 interaction through MR72 or mAb-548 (27).

Although the first-generation Trojan horse bsAbs afforded pan-ebolavirus neutralization, they lacked activity against more divergent filoviruses due to their reliance on FVM09, an ebolavirus-specific binder, for intracellular delivery with viral particles. Moreover, these bsAbs were susceptible to viral escape through a single point mutation in the FVM09 epitope, which abrogated their cellular internalization (27). Here, we overcame these liabilities by harnessing an internalizing cell-surface receptor, cation-independent mannose-6-phosphate receptor/insulin-like growth factor 2 receptor (CI-MPR/IGF2R) (28–32), for intracellular antibody delivery, instead of a viral epitope. We describe second-generation Trojan horse bsAbs that exploit two distinct CI-MPR/IGF2R-ligand interactions to internalize into cells and neutralize filovirus entry with enhanced potency and pan-filovirus breadth. This study expands the Trojan horse delivery mechanism to more fully exploit the therapeutic potential of antibodies against conserved but cryptic epitopes.

## Materials and Methods

### Antibody Expression and Purification

To generate IGF2-tagged bsAbs, the synthetic gene encoding the mature IGF2 sequence (Δ1-7,F26S,R37K,R40K) was subcloned into the pMAZ-IgH and pMAZ-IgL vectors and linked at the N-terminus to the variable domain of the heavy and light chains of mAbs MR72 and mAb-548 *via* a short amino acid linker “ASTKGP” or “TVAAP”, respectively. NPC2-tagged bsAbs were similarly subcloned as above. To express these bsAbs, pMAZ-IgH and pMAZ-IgL encoding each antibody were co-transfected into Freestyle™ 293-F cells, (ThermoFisher Scientific) using linear polyethylenimine (Polysciences). Cells were incubated in Freestyle™ 293 expression media, at 37°C with 8% CO_2_ in a shaking incubator for 6 days. Cells were pelleted and then the clarified supernatant was incubated with Protein A resin (1 ml packed resin per 600 ml clarified supernatant) for 2 h at 4°C. Antibodies were then purified using the gentle antibody elution system (ThermoFisher Scientific) per the manufacturer’s instructions. Eluted antibody was buffer-exchanged into Hepes buffer (200mM NaCl, 150mM HEPES[pH 7.4]) and concentrated using an Amicon centrifugal filter unit (Millipore Sigma) with a nominal molecular weight cutoff of 50 kDa.

### Protein Expression and Purification CI-MPR Domains 1-3 and 11-13

Synthetic genes encoding C-terminally hexahistidine tagged CI-MPR domains 1–3 (amino acids 36–466) and domains 11–13 (amino acids 1508–1992) or NPC2 with a Twin-Strep-tag were subcloned into linearized pHL-sec vectors using AgeI and KpnI restriction sites. Proteins were expressed in Freestyle™ 293-F cells by transient transfection as above. Clarified supernatants containing CI-MPR domains were incubated with Ni-NTA resin (1ml packed resin per 300ml supernatant) at 4°C for 2 h prior to collection into a column. Resin was washed with wash buffer [500mM NaCl, 20mM HEPES(pH 7.6)] and protein eluted with elution buffer [500mM Imidazole, 500mM NaCl, 20mM HEPES(pH 7.6)]. NPC2 was purified from clarified supernatants using IBA Strep-Tactin^®;^ Sepharose^®^ (IBA) according to the manufacturer’s protocol. All proteins were dialyzed overnight in PBS and subsequently concentrated using an Amicon centrifugal filter unit (Millipore Sigma) with a nominal molecular weight cutoff of 10 kDa. Protein purity was assessed by SDS-polyacrylamide gel electrophoresis and visualized by staining with Coomassie Brilliant Blue G-250.

### Preparation of NPC1 Loop C and EBOV GP_CL_

Soluble human NPC1 loop C was produced by IBT Bioservices as described previously (33). Ebola virus GPΔMuc [EBOV-Mayinga GP lacking the mucin-like domain (residues 312 to 462)] was expressed in *Drosophila melanogaster* S2 cells as previously described (14). EBOV GP_CL_ was produced by incubating trimeric GPΔMuc with thermolysin (Promega, V4001) in a 50:1 (protein:enzyme) ratio for approximately 18 h overnight at room temperature in 10 mM Tris-buffered saline [TBS; Tris-HCl(pH 7.5), 150 mM NaCl] containing 1 mM CaCl_2_. Trimeric EBOV GP_CL_ was purified by Superdex S200 size exclusion chromatography in TBS immediately following incubation.

### ELISAs for CI-MPR Domains

High-binding half-area 96-well ELISA plates (Corning) were incubated with 0.5 µg per well of CI-MPR domains 1–3 or 11–13 in PBS overnight at 4°C. Coated plates were blocked with blocking buffer (5% w/v nonfat dry milk in PBS) for 2 h at 37°C. A 3-fold dilution series of antibodies starting at 100 nM diluted in wash buffer (1% w/v nonfat dry milk in PBS) was incubated for 1 h at 37°C. Plates were washed 3 times with wash buffer. Anti-human IgG-HRP or anti-His tag-HRP secondary conjugates were diluted in wash buffer and incubated for 1 h at 37°C. Plates were washed with PBS and developed using Ultra-TMB (ThermoFisher) and quenched with 0.5M H_2_SO_4_. Absorbance was read at 450nm on a Cytation 5 cell imaging multi-mode reader (BioTek).

### BLI Assays

The OctetRed™ system (ForteBio, Pall LLC) was used to determine parental and bsAb binding properties. Anti-human Fc sensors were used for initial capture of IgG loading, and then measured for association to antigen at pH 5.5. Global fitting to a 1:1 binding model was used to estimate k_on_ (association rate constant), k_off_ (dissociation rate constant) and apparent K_D_^app^ (apparent equilibrium constant). Although data could be described accurately with a 1:1 model, given the bivalent nature of the antibody, and the trivalent nature of GP_CL_, we cannot rule out avidity effects and therefore report apparent KD.

### Cells

Human U2OS osteosarcoma cells were cultured in modified McCoy’s 5A medium (LifeTechnologies) supplemented with 10% fetal bovine serum (FBS; Atlanta Biologicals), 1% GlutaMAX (Life Technologies) and 1% penicillin/streptomycin (Life Technologies). Wildtype and cation-independent mannose-6-phosphate receptor (CI-MPR)–knockout human haploid Hap1 cells (Horizon Discovery, Cat# HZGHC006178c011) were cultured in Iscove’s modified Dulbecco’s medium (IMDM; ThermoFisher) and supplemented as above. Vero cells were maintained in high-glucose Dulbecco’s modified Eagle medium (DMEM; ThermoFisher) supplemented with 10% fetal bovine serum (FBS; Atlanta Biologicals), 1% GlutaMAX (Life Technologies) and 1% penicillin/streptomycin (Life Technologies). Human monocyte THP-1 cells (ATCC) were cultured in RPMI-1640 medium (ATCC, Cat #30-2001) supplemented with 10% FBS. All cells were maintained at 37°C, 5% CO_2_ in a humidified incubator.

### Viruses

Generation and propagation of recombinant vesicular stomatitis viruses (rVSVs) encoding enhanced green fluorescent protein (eGFP) in the first position and replacing VSV G with glycoproteins from EBOV/Mayinga (EBOV/H.sap-tc/COD/76/Yambuku-Mayinga), TAFV (TAFV/H.sap-tc/CIV/94/CDC807212), BDBV (BDBV/H.sap/UGA/07/But-811250), BOMV (BOMV/Mops condylurus/SLE/2016/PREDICT_SLAB000156), SUDV/Boneface (SUDV/C.por-lab/SSD/76/Boneface), RESTV (RESTV/M.fas-tc/USA/89/Phi89-AZ-1435), LLOV (LLOV/M.sch-wt/ESP/03/Asturias-Bat86) and an rVSV encoding an mNeongreen-phosphoprotein P (mNG-P) fusion protein bearing MARV GP (MARV/H.sap-tc/KEN/80/Mt. Elgon-Musoke) were previously described (2, 5, 27, 34–37). An rVSV encoding eGFP in the first position and MLAV GP (MLAV/Rousettus-wt/CHN/2015/Sharen-Bat9447-1; GenBank Accession no: KX371887) was cloned and rescued as above (2, 5, 27, 38–41).

### VSV Infection Assays

Dilution series of antibody or NPC2 were incubated on monolayers of human U2OS cells, wild type or CI-MPR–knockout human haploid Hap1 cells, or THP-1 cells for 2 h at 37°C prior to addition of pre-titrated amounts of rVSV-GP particles (MOI ~ 1 infectious unit (IU) per cell). For infection assays using THP-1 cells, cells were treated with 20ng/ml phorbol 12-myristate 13-acetate (PMA) (Millipore Sigma, Cat#P1585) for 72 h and media was exchanged prior to antibody incubation (42). Viral infectivities were measured by automated enumeration of eGFP^+^ or mNG^+^ cells using a Cytation 5 reader at 12-14 h post-infection. Data was subjected to non-linear regression analysis to extract half maximal inhibitory concentration (IC_50_) values (4-parameter, variable slope sigmoidal dose-response equation; GraphPad Prism). Relative IC_50_ values were calculated for all curves with sigmoidal curves and absolute IC_50_ values were calculated for curves with ill-defined plateaus.

### pHrodo Red Labeling of Antibodies and Flow Cytometry

Parental antibodies and bsAbs were covalently labeled with pH-sensitive pHrodo Red succinimidyl ester (Thermo Fisher Scientific) according to the manufacturer’s instructions. Antibodies were incubated with 10-fold molar excess of pHrodo Red succinimidyl ester for 1 h at room temperature. Excess unconjugated dye was removed using PD-10 desalting columns (GE Healthcare). pHrodo Red-labeled antibodies were exchanged into HEPES buffer and concentrated in an Amicon Ultra centrifugal filter unit with a nominal molecular weight cutoff of 30 kDa. Antibody concentration and degree of labeling was determined according to the manufacturer’s instructions.

Pre-chilled confluent human U2OS cell monolayers were incubated with the pHrodo Red-labeled parental and bsAbs (50 nM) at either 4°C to prevent internalization or 37°C to allow internalization for 30 min. Cells were returned to ice and any unbound antibody was removed by washing with cold PBS. Cells were harvested using cold trypsin-EDTA for 10 min. Cells were washed with cold PBS prior to filtering through a mesh strainer. Single cells were analyzed for pHrodo Red fluorescence on a BD LSRII flow cytometer and FlowJo software.

### CellTiter-Glo Assay

A dilution series of antibodies were incubated on monolayers of U2OS cells for 14 h at 37°C. A 1:1 ratio of CellTiter-Glo reagent (Promega) to cell culture media was added per well. Contents were mixed for 2 minutes on an orbital shaker then incubated at room temperature for 10 minutes before luciferase activity was read using a Cytation 5 reader.

### Filipin Staining

U2OS cells were seeded on fibronectin-coated coverslips. Cells were incubated with either media alone, U18666A (10 µM, Millipore Sigma), or antibody (1 nM or 350 nM) at 37°C for 14 h prior to fixing. Cells were washed with PBS prior to incubation with filipin III (Millipore Sigma) for 1 h at room temperature, washed again with PBS, and then mounted onto slides with Prolong (Thermo Fisher). Slides were imaged using a Zeiss Axio Observer inverted microscope with a 40x objective.

### Authentic Filovirus Infections

A dilution series of antibodies were incubated on monolayers of Vero E6 or U2OS cells for 2 h at 37°C prior to addition of pre-titrated amount of Ebola virus/H.sapienstc/COD/1995/Kikwit-9510621 (EBOV/Kik-9510621; ‘EBOV-Zaire 1995’) or Marburg virus/*H.sapiens*tc/DEU/1967/Hesse-Ci67. At 48 h post-infection, cells were fixed with formalin, and blocked with 1% bovine serum albumin. EBOV-infected cells, MARV-infected cells and uninfected controls were incubated with either EBOV GP-specific mAb KZ52 or MARV GP-specific mAb 9G4, respectively. Cells were washed with PBS prior to incubation with either goat anti-human IgG or goat anti-mouse IgG conjugated to Alexa Fluor 488 (Invitrogen). Cells were counterstained with Hoechst stain (Invitrogen), washed with PBS and stored at 4°C. Infected cells were quantitated by fluorescence microscopy and automated image analysis using an Operetta high content device (Perkin Elmer) and the image analysis Harmony software, as previously described (27).

## Results

### Design and Biochemical Characterization of NPC2- and IGF2-Tagged Trojan Horse bsAbs

**Figure 1:**
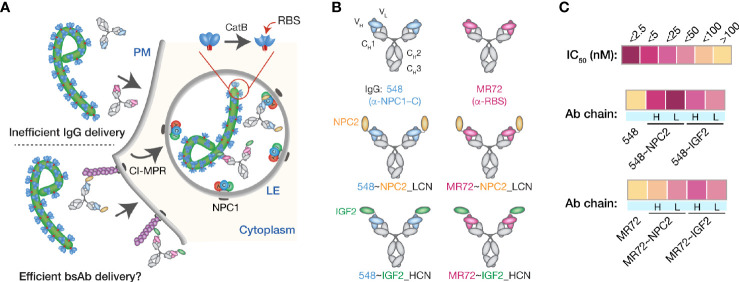
Design and initial evaluation of bispecific antibodies combining CI-MPR ligands and mAbs blocking the endo/lysosomal filovirus GP:NPC1 interaction. **(A)** A hypothetical mechanism for delivery of NPC2- and IGF2-tagged bsAbs (bottom) but not parental mAbs (top) to sites of GP_CL_:NPC1 interaction in late endosomal/lysosomal (LE) compartments. **(B)** Schematic representations of a subset of the antibodies tested in this study. Top row: parental mAbs, NPC1 domain C (NPC1–C)-specific mAb-548 and viral glycoprotein receptor-binding site (RBS)-specific mAb MR72. Middle row: NPC2-tagged bsAbs. NPC2 was fused to the N–terminus of the light chain (LCN) of mAb-548 and MR72. Bottom row: IGF2-tagged bsAbs. IGF2 was fused to the N–terminus of the heavy chain (HCN) of mAb-548 and MR72. **(C)** Heat map showing neutralizing IC_50_ values against rVSV-EBOV GP for the antibody panel. For curves that did not cross the 50% threshold, IC_50_ values were considered >100 nM.

We explored a multifunctional cell-surface receptor, the cation-independent mannose-6-phosphate receptor/insulin-like growth factor 2 receptor (hereafter, CI-MPR), as an alternative endo/lysosomal delivery strategy for mAbs targeting the filovirus GP_CL_:NPC1 interaction. CI-MPR is a ~300-kDa Type I membrane glycoprotein comprising a large extracellular domain with distinct binding sites for multiple ligands, a transmembrane region, and a cytoplasmic tail that regulates receptor internalization, endocytic trafficking, and recycling (43). Mannose-6-phosphate (M6P) and insulin-like growth factor 2 (IGF2) are the best characterized CI-MPR ligands and both interactions have been successfully exploited for endo/lysosomal delivery of recombinant cargo molecules (29–31, 44–46). Many lysosomal enzymes naturally undergo mannose-6-phosphorylation, affording their CI-MPR–mediated extracellular retrieval and intracellular delivery as enzyme replacement therapies for lysosomal storage disorders (34, 47–49). Here, we sought to exploit one such lysosome-resident protein, a 132-amino acid sterol-binding protein Niemann-Pick C2 (NPC2) (50, 51), to deliver mAb cargoes to late endosomes and lysosomes (**Figure 1A**). Accordingly, we fused NPC2 to mAbs MR72 and mAb-548 in two configurations (to the *N*–terminus of the IgG heavy or light chain) to create a panel of bsAbs. This panel was screened for neutralization potency against a surrogate vesicular stomatitis virus bearing EBOV GP (rVSV-EBOV GP) (39). We identified two highly active candidates mAb-548 and MR72 bearing NPC2 at the *N*–termini of their light chains (548~NPC2_LCN and MR72~NPC2_LCN, respectively; hereafter 548~NPC2 and MR72~NPC2) (**Figures 1B, C** and **Supplementary Figures S1A, B**).

To address concerns with inconsistent mannose-6-phosphorylation (M6P) of lysosomal proteins during manufacturing, LeBowitz and colleagues described a glycosylation-independent lysosomal targeting (GILT) approach in which recombinant cargoes were fused to the 61-amino acid mature IGF2 peptide sequence (30, 44). These proteins could be captured and internalized through IGF2:CI-MPR binding, thus bypassing the need for M6P. To test the efficacy of this approach for our mAbs, we generated a panel of IGF2-tagged bsAbs and down-selected them as above to identify two molecules bearing IGF2 at the *N–*termini of the mAb heavy chains (548~IGF2_HCN and MR72~IGF2_HCN, respectively; hereafter 548~IGF2 and MR72~IGF2) (**Figures 1B, C** and **Supplementary Figures S1A, B**).

**Figure 2:**
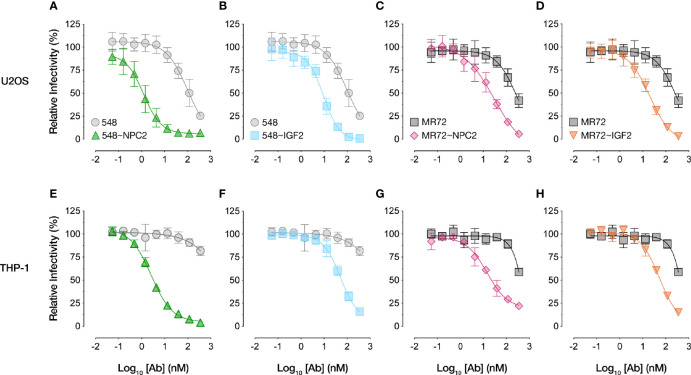
NPC2- and IGF2-tagged bsAbs neutralize VSV-EBOV in multiple cell types. Neutralization curves of bsAbs and parental mAbs against rVSV-EBOV GP in **(A-D)** human U2OS osteosarcoma cells or **(E-H)** human THP-1 cells differentiated into macrophage-like cells. Infection was measured by automated counting of eGFP+ cells and normalized to infection in absence of antibody. Means ± SD are shown for 4–8 replicates from 2–4 independent experiments.

**Figure 3:**
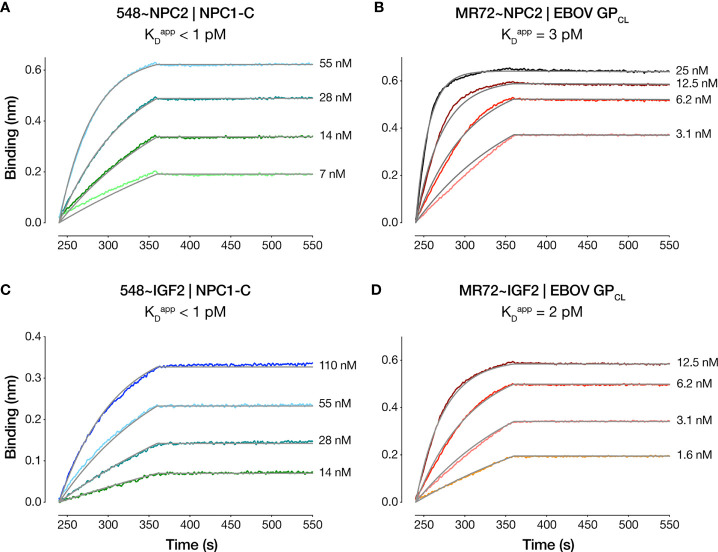
Antibody-antigen binding profiles of NPC2- and IGF2-tagged bsAbs. Kinetic binding curves for NPC2- and IGF2-tagged bsAb:antigen interactions were determined by BLI. **(A)** 548~NPC2 and **(C)** 548~IGF2 were loaded onto probes and dipped into analyte solutions containing the indicated concentrations of NPC1-C. **(B)** MR72~NPC2 and **(D)** MR72~IGF2 were loaded onto probes and dipped into the indicated concentrations of analyte solutions containing EBOV GP_CL_. Grey lines indicate curve fits to a 1:1 binding model. See **Table 1** for kinetic binding constants.

We compared the dose-dependent antiviral activities of the down-selected bsAbs in the rVSV-EBOV GP infection assay in two cell types: U2OS human osteosarcoma cells and THP-1 human monocyte-like cells differentiated to macrophage-like cells with PMA. All four bsAbs afforded neutralization with low-nM IC_50_ values and with ~10–100-fold greater potency than their parental IgGs, although the NPC2-tagged bsAbs were relatively more potent than their IGF2-tagged counterparts (**Figure 2**). Importantly, only the bsAbs afforded complete neutralization. Consistent with their enhanced neutralizing activity, our top four bsAbs retained high-affinity binding to their respective endo/lysosomal target antigens at acid pH, as determined by biolayer interferometry (BLI) (**Figure 3** and **Table 1**). NPC2- and IGF2-tagged bsAbs displayed similar binding activities, indicating that the reduced activity of the latter is not a consequence of antigen:Ab binding penalties exacted by the NPC2 or IGF2 tag.

**Table 1:** Kinetic binding constants for Ab:antigen interaction determined by BLI.

Antibody	Antigen	pH	Average K_D_^app^ (pM)^a^
MR72	EBOV GP_CL_	5.5	2±1
MR72~NPC2	EBOV GP_CL_	5.5	3±4
MR72~IGF2	EBOV GP_CL_	5.5	2±2
548	Human NPC1-C	5.5	7±10
548~NPC2	Human NPC1-C	5.5	<1
548~IGF2	Human NPC1-C	5.5	<1

^(a)^K_D_^app^, apparent equilibrium dissociation constant. Mean±SD determined from three independent experiments

### IGF2- and NPC2-Tagged bsAbs Are Targeted to Distinct, Ligand-Specific Domains in CI-MPR

**Figure 4:**
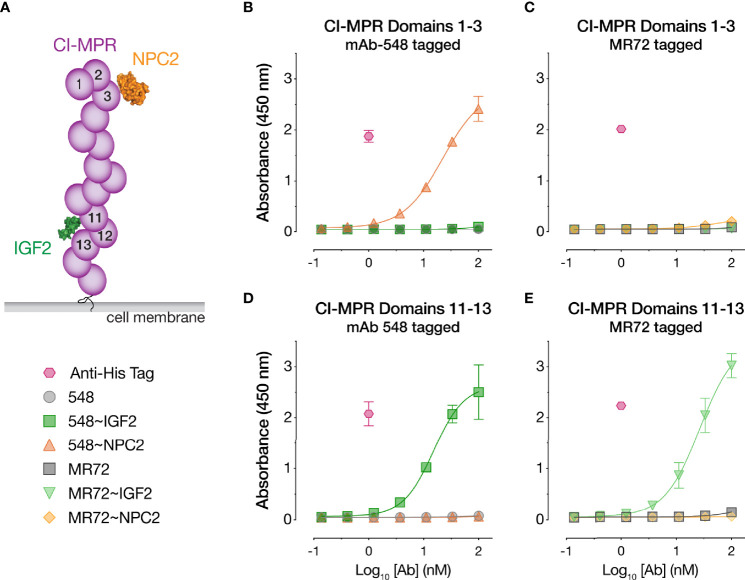
CI-MPR domain binding profiles of NPC2- and IGF2-tagged bsAbs. **(A)** Schematic of CI-MPR recognition by NPC2 and IGF2. **(B, C)** ELISA reactivity of the indicated bsAbs and parental mAbs against CI-MPR domains 1–3. **(D, E)** ELISA reactivity of the indicated bsAbs and parental mAbs against CI-MPR domains 11–13. Means ± SD are shown for 6 replicates from 2 independent experiments.

The CI-MPR ectodomain contains 15 ~150-amino acid ‘mannose 6-phosphate receptor homology’ domains (52). Domain 11 recognizes IGF2 (35, 53), and domains 3, 5, 9, and 15 recognize M6P (52) (**Figure 4A**). To begin to investigate the mechanisms of action of our Trojan horse bsAbs, we assessed their binding to recombinant, purified CI-MPR domains (**Supplementary Figures S1C, D**) by ELISA. Both IGF2-tagged bsAbs bound to a CI-MPR domain 11–13 construct predicted to recognize IGF2 but not M6P, whereas their NPC2-tagged and parental IgG counterparts did not (**Figures 4D, E**). Conversely, only 548~NPC2 bound to a CI-MPR domain 1–3 construct predicted to recognize M6P but not IGF2 (**Figure 4B**). Unexpectedly, MR72~NPC2 showed no detectable binding to domain 1–3 in the ELISA (**Figure 4C**), suggesting that the MR72 light chain may sterically hinder CI-MPR recognition by NPC2 but not by the much smaller IGF2 peptide. We hypothesize that reduced CI-MPR binding by MR72~NPC2 accounts for its ~10-fold reduced antiviral potency relative to 548~NPC2 (**Figures 1C** and **2A, C, E, G**).

### NPC2-tagged bsAbs Are Dependent on CI-MPR for Antiviral Activity

**Figure 5:**
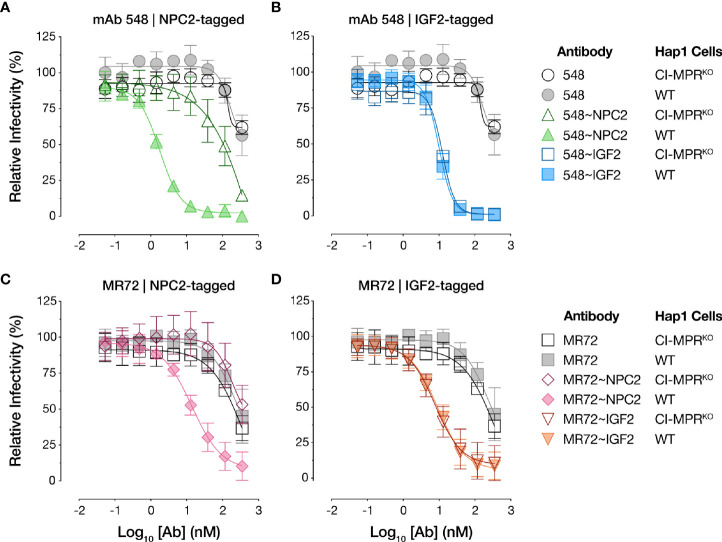
NPC2- but not IGF2-tagged bsAbs lose neutralizing activity in CI-MPR knockout cells. Capacity of **(A, C)** NPC2-tagged and **(B, D)** IGF2-tagged bsAbs to neutralize rVSV-EBOV GP infection in isogenic haploid cell lines replete with or genetically deficient in CI-MPR (Hap1 and Hap1-CI-MPR^KO^, respectively). Infection was measured by automated counting of eGFP^+^ cells and normalized to infection in absence of antibody. Means ± SD are shown for 5–8 replicates from 2–3 independent experiments.

To test if the bsAbs require cellular CI-MPR for their antiviral activity, we next evaluated their capacity to neutralize rVSV-EBOV GP infection in isogenic haploid cell lines replete with or genetically deficient in CI-MPR (Hap1 and Hap1-CI-MPR^KO^, respectively). Both NPC2-tagged bsAbs suffered an almost complete loss in activity in Hap1-CI-MPR^KO^ cells relative to the Hap1 cells, indicating that their antiviral activity was largely dependent on CI-MPR (**Figures 5A, C**). By contrast, the IGF2-tagged bsAbs were insensitive to CI-MPR loss (**Figures 5B, D**), indicating the existence of CI-MPR-independent pathways for their internalization into target cells (see below).

### NPC2 and IGF2 Tags Afford mAb Internalization Into Acidic Intracellular Compartments

**Figure 6:**
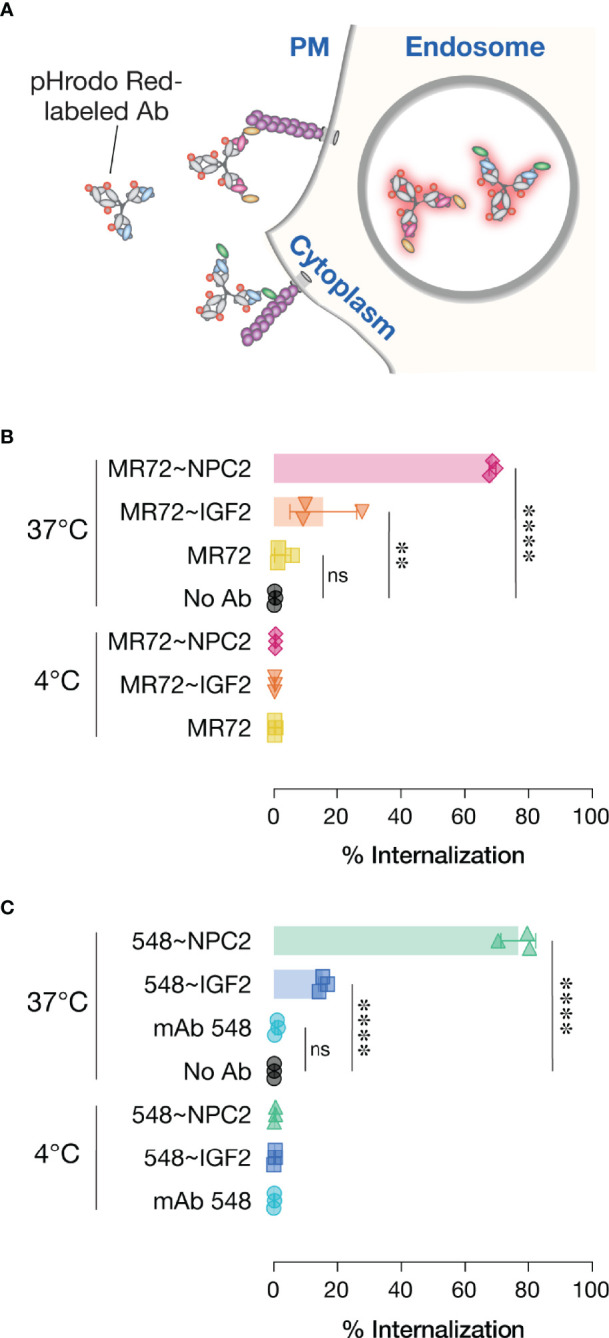
Cellular internalization of Trojan horse bsAbs. **(A)** Schematic of pHrodo Red-labeled antibody internalization experiment. **(B, C)** Labeled antibodies were incubated with cells at either 4°C to prevent internalization or 37°C to allow internalization prior to analysis by flow cytometry. Data points represent the percentage of pHrodo Red positive cells; bars represent means ± SD from 3 independent experiments. Group means (bars) were compared using one-way ANOVA with Turkey’s multiple comparisons test. (****P < 0.0001; **P < 0.01; ns, not significant).

The basis of our strategy was that the NPC2- and IGF2-tags would mediate cellular receptor-mediated internalization and delivery of bsAbs to their endo/lysosomal sites of antiviral action. To evaluate this premise, we conjugated an acid-dependent fluorescent dye, pHrodo Red™, to each bsAb and its parent IgG, and measured the capacity of these conjugates to undergo temperature-dependent delivery to acidic intracellular compartments (**Figure 6A**), as described previously (27). As expected, no increases in single cell-associated pHrodo Red fluorescence was observed by flow cytometry with any of the mAbs or bsAbs following their incubation with cells for 30 min at 4°C. Similar results were obtained with the parent mAbs after 30 min at 37°C, as we observed previously (27). By contrast, considerable proportions of all four bsAbs were internalized under the same conditions, although the NPC2-tagged bsAbs appeared to undergo uptake more efficiently than did the IGF2-tagged molecules (**Figures 6B, C**). These findings support a model in which tag-mediated interaction with CI-MPR (NPC2) and/or other cell-surface receptors (IGF2) enhances bsAb internalization and delivery to NPC1-bearing compartments.

### NPC2-Tagged bsAbs Do Not Inhibit Viral Entry Through the Direct Action of NPC2

**Figure 7:**
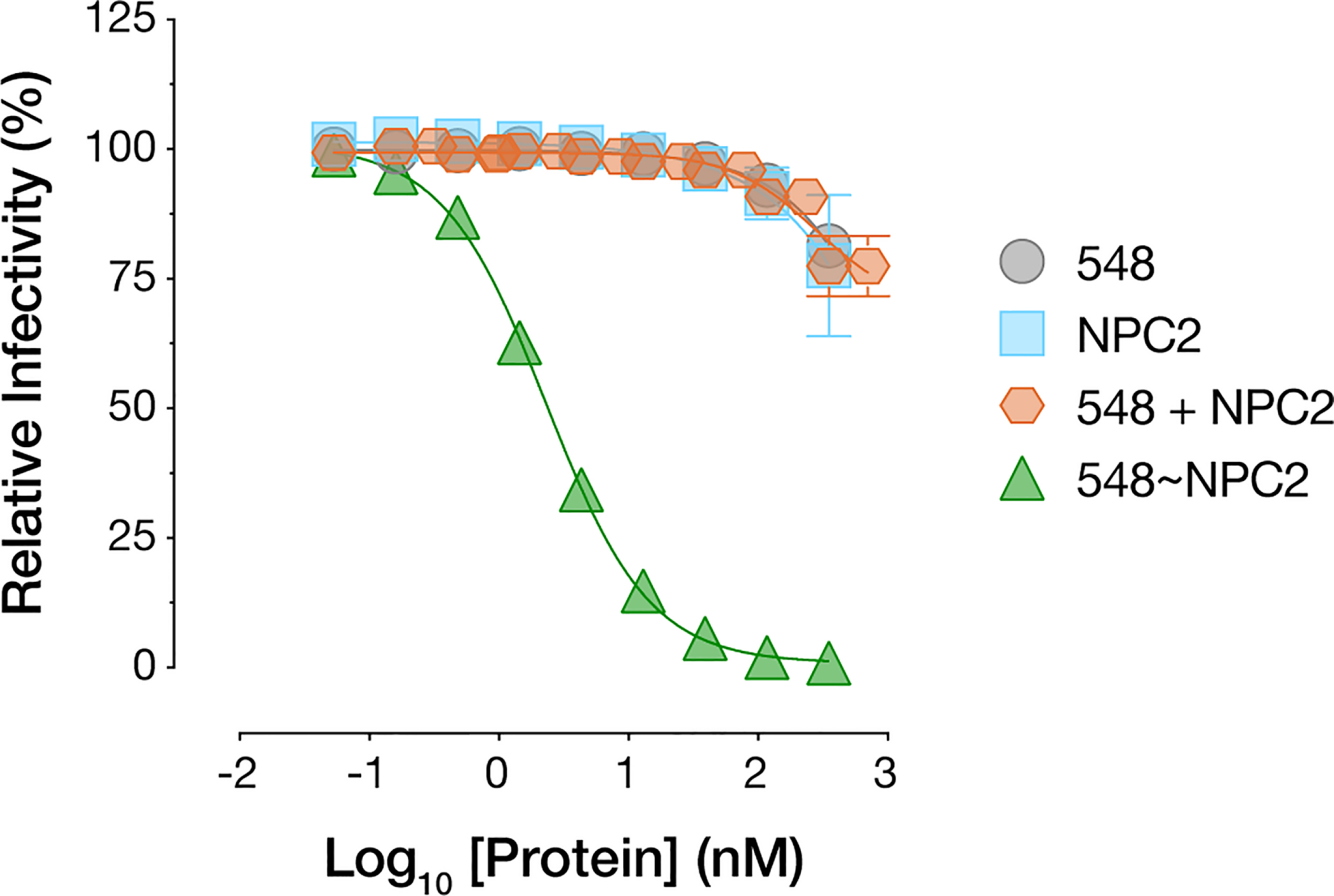
NPC2 does not directly block viral entry. mAb-548, NPC2, equimolar combination of mAb-548 and NPC2, and 548~NPC2 bsAb were incubated with U2OS osteosarcoma cells for 2 h at 37°C prior to exposure to rVSV-EBOV GP. Infection was measured by automated counting of eGFP^+^ cells and normalized to infection in absence of antibody or protein. Means ± SD are shown for four replicates from two independent experiments.

The superior cell-internalizing activity of the NPC2-tagged bsAbs relative to their IGF2-tagged counterparts suggested an explanation for the former’s enhanced antiviral potency. However, we also considered an additional hypothesis to explain these results. Specifically, NPC2 engages NPC1 as part of their interdependent function in cellular cholesterol trafficking and does so *via* a protein-protein interface that overlaps the filovirus GP_CL_:NPC1 interface (54, 55). Thus, it remained possible that NPC2-tagged bsAbs compromise virus-receptor association directly through both mAb- and NPC2-dependent mechanisms. To address this possibility, we incubated cells with purified recombinant NPC2 (**Supplementary Figure S1E**), separately and in combination with mAb-548, and then exposed the cells to rVSV-EBOV GP. Neither treatment afforded a significant enhancement in viral neutralization relative to mAb-548 alone (**Figure 7**). Further, only bsAbs bearing the NPC1-binding mAb-548 inhibited NPC1 function as measured by lysosomal accumulation of free cholesterol (56, 57), regardless of whether mAb-548 was fused to NPC2 or IGF2 (**Supplementary Figure S2A**). These results are consistent with a scenario in which NPC2 acts only to mediate endo/lysosomal delivery of the bsAbs and not as part of their antiviral payload.

Finally, we note that NPC1 inhibition was only observed following long-term treatment with high doses of bsAbs and did not compromise cell metabolic health or induce cytotoxicity (**Supplementary Figure S2B**).

### NPC2- and IGF2-Tagged bsAbs Block Entry Mediated by All Known Filovirus GP Proteins and Neutralize Infection by Two Divergent Authentic Filoviruses

**Figure 8:**
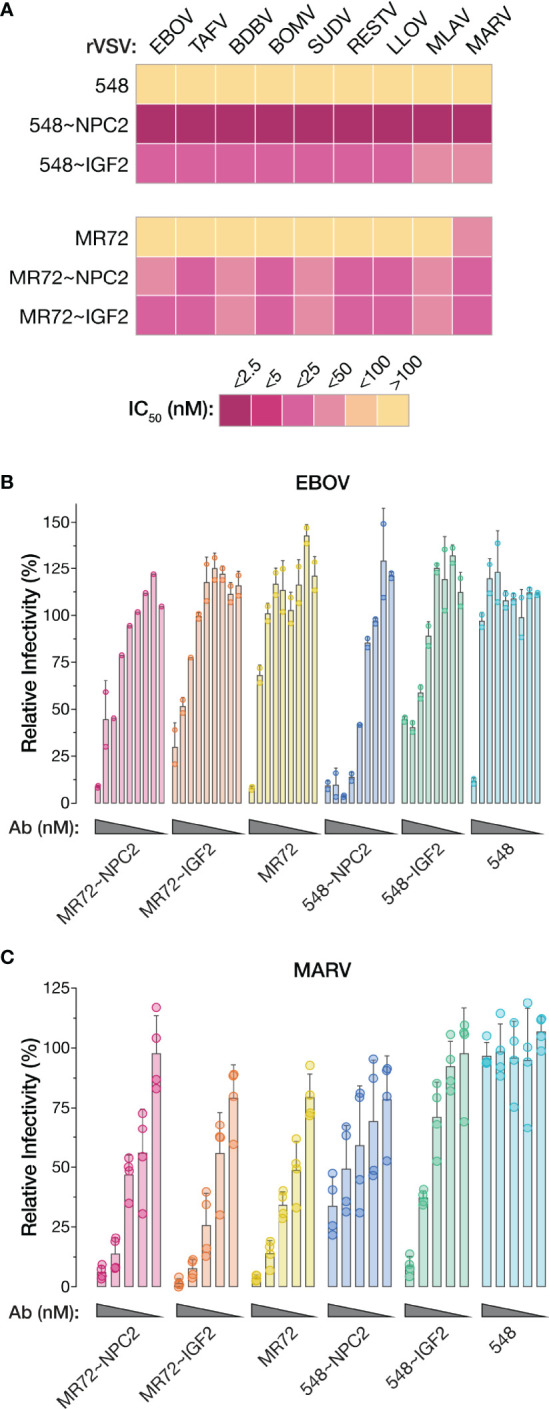
NPC2- and IGF2-tagged bsAbs afford pan-filovirus entry inhibition. **(A)** Heat map showing neutralizing IC_50_ values for parental mAbs and NPC2- and IGF2-tagged bsAbs against all rVSVs bearing filovirus glycoproteins. IC_50_ values were calculated from neutralization curves from 4–6 replicates from 2–3 independent experiments. For curves that did not cross the 50% threshold, IC_50_ values were considered >100 nM. EBOV, Ebola virus; TAFV, Tai Forest virus; BDBV, Bundibugyo virus; BOMV, Bombali virus; SUDV, Sudan virus; RESTV, Reston virus; LLOV, Lloviu virus; MLAV, Mengla virus; MARV, Marburg virus. **(B, C)** Neutralization of authentic **(B)** EBOV and **(C)** MARV by parental mAbs and NPC2- and IGF2-tagged bsAbs. Means ± SD are shown for 2–4 replicates from 1–2 independent experiments.

The GP_CL_:NPC1 interaction interface is highly conserved and required for all known mammal-infecting filoviruses, including both known and potential zoonotic threats (2, 5, 6, 19–22, 24). Given our evidence that the NPC2- and IGF2-tagged bsAbs could target this interaction to inhibit EBOV GP-dependent entry, we reasoned that they may possess broad anti-filovirus activity. Accordingly, we tested the bsAb panel against rVSVs bearing each of the filovirus GPs (ebolaviruses: BDBV, TAFV, BOMV, RESTV; SUDV, cuevavirus: LLOV, dianlovirus: MLAV, marburgvirus: MARV). All four bsAbs afforded substantial enhancements in neutralization potency relative to their parental IgGs against all of the rVSVs (**Figures 8A** and **Supplementary Figures S3, S4**). The MR72-containing bsAbs alone provided only limited gains in neutralizing activity against rVSV-MARV GP (**Figures 8A**, **Supplementary Figures S3B, S4C, D**), likely reflecting MR72’s capacity to recognize and block the extracellular form of MARV GP but not of other filovirus GPs (27, 58). Finally, and importantly, these findings were concordant with the capacity of all four bsAbs to neutralize two divergent, authentic human disease-causing filoviruses, EBOV and MARV (**Figures 8B, C**). We conclude that the endo/lysosomal filovirus-receptor interaction represents a universal Achilles heel that can be targeted to develop pan-filovirus entry inhibitors.

## Discussion

Currently approved antibody therapeutics for filovirus infections are limited to the treatment of EBOV disease and do not neutralize or protect against other mammal-infecting filoviruses with known or suspected zoonotic potential (7, 8, 11–13). A number of recent studies have sought to address this unmet need for broadly protective antibody-based treatments. Wec, Bornholdt, and co-workers screened a large panel of human mAbs to identify broadly neutralizing antibodies (bnAbs) targeting conserved ebolavirus GP epitopes outside the RBS and developed them into a cocktail, MBP134, with pan-ebolavirus breadth and protective efficacy against EBOV, BDBV, and SUDV in nonhuman primates (14, 15, 40). Similar approaches were employed to isolate other ebolavirus-specific bnAbs (59–62). Multispecific antibodies with enhanced antiviral breadth and cross-protective efficacy have also been engineered and evaluated in rodent models of filovirus challenge. nAbs specific for different ebolaviruses and MARV were successfully combined into broadly neutralizing bispecific and trispecific antibodies (63, 64). In the distinct Trojan horse approach, bsAbs were generated by linking a pan-ebolavirus non-neutralizing mAb, FVM09, with either of two mAbs targeting cryptic endosomal epitopes at the GP_CL_:NPC1 interface (27). Unlike the preceding multispecific antibodies, the Trojan horse bsAbs not only possessed pan-ebolavirus breadth but also exhibited ‘obligate’ activity, in that they required both antibody combining sites to neutralize infection. Here, we describe a modified Trojan horse bsAb strategy that leverages an internalizing host cell receptor for endo/lysosomal delivery to neutralize entry mediated by all known filovirus glycoproteins.

Classical approaches to promote the membrane passage and intracellular delivery of large biomolecules such as antibodies have largely used chemical (e.g., liposomes) and physical agents (e.g., electroporation) [reviewed in (65)]. While these methods remain in wide use, ‘cell-penetrating peptides’ (CPPs) have also been increasingly employed for this purpose (65–68). Transbodies—antibodies coupled to a CPP—were shown to suppress hepatitis B virus replication (66) and inhibit EBOV VP35’s functions in viral genome replication and host interferon antagonism (68). CPPs have been used to target antiviral molecules not only to the cytoplasm, but also to endosomal compartments—a membrane fusion-inhibitory peptide from EBOV GP could block viral entry when targeted to endosomes *via* a CPP (69). Conjugation of such glycoprotein-derived peptides to cholesterol also afforded enhanced antiviral activity through both cell surface and endosomal modes of action (70–75).

Another strategy for intracellular targeting instead leverages native cell surface receptor-ligand interactions and has found broad application in enzyme replacement therapies for lysosomal storage diseases (30, 32, 44, 76–78). These treatments take advantage of endogenous interactions between oligosaccharide-modified lysosomal enzymes and cell-surface receptors that mediate enzyme trafficking, retrieval, and clearance, with the interaction between enzyme-linked mannose-6-phosphate and CI-MPR playing a key role (28, 32, 44, 79). Delivery of the payload is accomplished through its receptor-mediated internalization and trafficking to late endo/lysosomal compartments. Here, we show that the mannose-6-phosphorylated lysosomal protein NPC2 could mediate the delivery of mAbs to their intracellular sites of action in a CI-MPR–dependent manner. This is, to our knowledge, the first application of a lysosomal protein for endo/lysosomal targeting of a heterologous therapeutic molecule.

Because recombinant lysosomal enzymes display both enzyme- and batch-dependent variations in binding affinity for CI-MPR, a variation of the above strategy instead targets CI-MPR through its distinct IGF2-binding site by fusing the payload lysosomal enzyme to the mature IGF2 peptide sequence (44). We found that the IGF2 tag could also effectively deliver mAbs targeting the GP_CL_:NPC1 interface. CI-MPR was dispensable for the biological activity of the IGF2-tagged bsAbs, consistent with the capacity of IGF2 to bind other receptors, including insulin receptor (IR) and insulin-like growth factor 1 receptor (IGF1R). IGF2 uses different surfaces to interact with CI-MPR and IGF1R, and point mutations in IGF2 differentially affect its capacity to recognize these receptors (36, 37, 80–83), suggesting that receptor-selective variants could be engineered for use with Trojan horse bsAbs and other intracellular targeting applications. Given that the IGF2 tag was less effective at mediating bsAb internalization than the NPC2 tag, the engineering of such receptor-optimized mutant IGF2s with potentially fewer “off-target” binding partners may be warranted as part of the development of next-generation Trojan horse bsAbs.

One potential issue associated with therapeutics targeting virus-host interactions is toxicity caused by drug-mediated blockade of host factor function. Here, we observed that longer incubations of cells with high doses of the mAb-548–containing bsAbs inhibited NPC1’s cholesterol trafficking function, as evidenced by the endosomal accumulation of free cholesterol. By contrast, the virus-directed MR72-containing bsAbs did not inhibit NPC1 function in this assay, providing evidence that NPC1 inhibition is associated with mAb-548:NPC1 binding and not with the CI-MPR-based endo/lysosomal targeting modalities. Although none of our bsAbs induced cellular cytotoxicity, further investigation of mAb-548’s activity against NPC1’s host function in animal models is warranted. However, we note the temporary inhibition of NPC1 caused by therapeutic administration of mAb-548–containing bsAbs is unlikely to be a concern given the rapid course of filovirus disease.

Finally, recent work points to the broader utility of the endo/lysosomal delivery strategy we describe herein. Specifically, mAbs conjugated to synthetic M6P-containing glycopeptides were used to target secreted and membrane-associated proteins for lysosomal degradation *via* CI-MPR (84). We propose that NPC2 and IGF2 tags could provide alternative modalities to remove both cellular and viral biomolecules from circulation as part of therapeutic applications.

In sum, we demonstrate that bsAbs engineered to exploit host cell CI-MPR for endo/lysosomal targeting can unlock the full antiviral potential of mAbs that recognize the conserved, but cryptic site of vulnerability at the filovirus-receptor interface. These are the first single antibody-based molecules with pan-filovirus GP neutralization activity, and they warrant evaluation as therapeutics in animal models of filovirus challenge.

## Data Availability Statement

The original contributions presented in the study are included in the article/**Supplementary Material**. Further inquiries can be directed to the corresponding authors.

## Author Contributions

Conceptualization: ASW, AZW, JD, JL, and KC. Methodology: ASW, EN, AZW, AH, MS, AK, EM, RJ, JT, JD, JL, and KC. Investigation: ASW, AZW, EN, AH, and AK. Formal analysis: ASW and KC. Resources: EM and RJ. Writing—original draft: ASW, KC, and JL. Writing—review and editing: All authors. Visualization: ASW and KC. Supervision: JD, AH, KC, and JL. Funding acquisition: KC, JL, and JD. All authors contributed to the article and approved the submitted version.

## Funding

This research was partially supported by the U.S. National Institutes of Health (NIH) grants R01A1134824 (to KC) and R01AI125426 (to JL). ASW was partially supported by the NIH training grant T32AI070117 in Geographic Medicine and Emerging Infections to Albert Einstein College of Medicine.

## Author Disclaimer

Opinions, conclusions, interpretations, and recommendations are those of the authors and are not necessarily endorsed by the U.S. Army. The mention of trade names or commercial products does not constitute endorsement or recommendation for use by the Department of the Army or the Department of Defense.

## Conflict of Interest

KC is a member of the scientific advisory boards of Integrum Scientific, LLC, Biovaxys Technology Corp, and the Pandemic Security Initiative of Celdara Medical, LLC, and he has consulted for Axon Advisors, LLC. JL is a consultant for Celdara Medical, LLC. Author JT was employed by company Mapp Biopharmaceutical.

The remaining authors declare that the research was conducted in the absence of any commercial or financial relationships that could be construed as a potential conflict of interest.

## Publisher’s Note

All claims expressed in this article are solely those of the authors and do not necessarily represent those of their affiliated organizations, or those of the publisher, the editors and the reviewers. Any product that may be evaluated in this article, or claim that may be made by its manufacturer, is not guaranteed or endorsed by the publisher.

## Supplementary Material

Supplementary Figure S1 Biochemical characterization of antibodies and purified proteins. Purified antibodies **(A)** 548, 548~IGF2_HCN and 548~NPC2_LCN and **(B)** MR72, MR72~IGF2_HCN and MR72~NPC2_LCN were resolved by SDS-PAGE under reducing conditions. HCN, IGF2 or NPC2 fused to the N-terminus of the IgG heavy chain. LCN, IGF2 or NPC2 fused to the N-terminus of the IgG light chain. IgG heavy (H) and light (L) chains were visualized by Coomassie Brilliant Blue staining. Purified **(C)** CI-MPR Domains 1-3, **(D)** CI-MPR Domains 11-13, **(E)** NPC2 proteins were resolved by SDS-PAGE under nonreducing (NR) and reducing (R) conditions and visualized by Coomassie Brilliant Blue staining.

Supplementary Figure S2 mAb-548–containing bsAbs cause endo/lysosomal free cholesterol accumulation but do not affect cell viability. **(A)** Filipin staining of U2OS cells treated with the indicated antibodies (1 nM or 350 nM) or U18666A (positive control (54); 10 µM) for 14 h. **(B)** U2OS cell viability was determined by CellTiter-Glo assay following incubation with the indicated antibodies (1 nM or 350 nM) for 14 h. Means ± SD are shown for 4 replicates from 2 independent experiments.

Supplementary Figure S3 Breadth of neutralization by Trojan horse bsAbs. Neutralization curves for **(A)** the mab-548 Trojan horse bsAb panel and **(B)** the MR72 Trojan horse bsAb panel against rVSVs bearing filovirus glycoproteins. IC_50_ values were calculated from curves for **Figure 8A**. Means ± SD are shown for 4–6 replicates from 2–3 independent experiments. EBOV, Ebola virus; TAFV, Tai Forest virus; BDBV, Bundibugyo virus; BOMV, Bombali virus; SUDV, Sudan virus; RESTV, Reston virus; LLOV, Lloviu virus; MLAV, Mengla virus; MARV, Marburg virus.

Supplementary Figure S4 NPC2- and IGF2-tagged bsAbs neutralize VSV-MARV in THP-1 cells. Neutralization activity of **(A-B)** mAb-548–containing bsAbs and **(C-D)** MR72-containing bsAbs against rVSV-MARV GP in differentiated THP-1 cells. Infection was measured by automated counting of eGFP+ cells and normalized to infection in absence of antibody. Means ± SD are shown for 4 replicates from 2 independent experiments.
