# Supplementary figures and images for: Two Distinct Lysosomal Targeting Strategies Afford Trojan Horse Antibodies With Pan-Filovirus Activity
Source: Front Immunol. 2021 Oct 14;12:729851. doi: 10.3389/fimmu.2021.729851 (PMC8551868; doi:10.3389/fimmu.2021.729851)

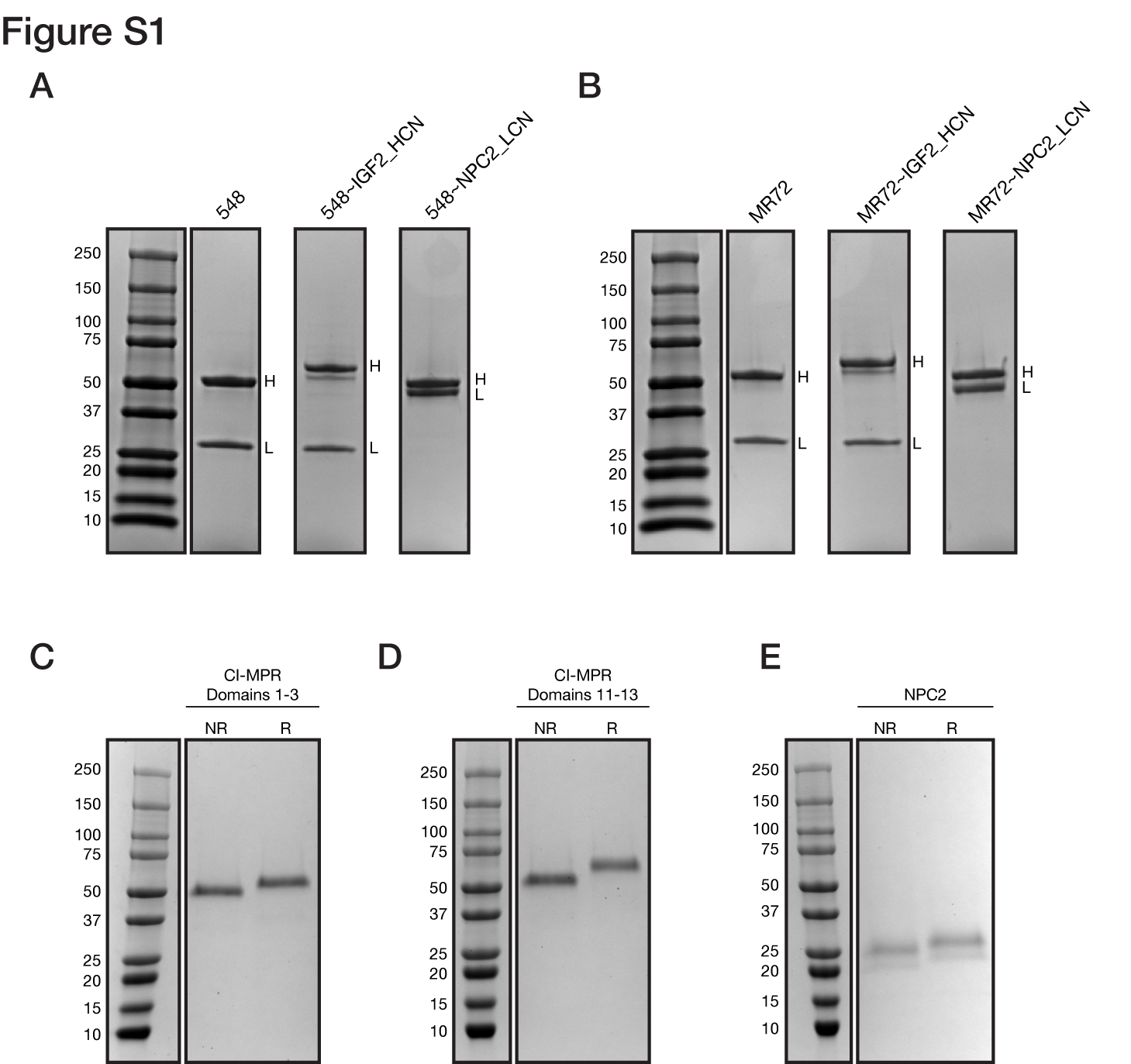

Supplement: Supplementary Figure S1 — Biochemical characterization of antibodies and purified proteins. Purified antibodies (A) 548, 548~IGF2_HCN and 548~NPC2_LCN and (B) MR72, MR72~IGF2_HCN and MR72~NPC2_LCN were resolved by SDS-PAGE under reducing conditions. HCN, IGF2 or NPC2 fused to the N-terminus of the IgG heavy chain. LCN, IGF2 or NPC2 fused to the N-terminus of the IgG light chain. IgG heavy (H) and light (L) chains were visualized by Coomassie Brilliant Blue staining. Purified (C) CI-MPR Domains 1-3, (D) CI-MPR Domains 11-13, (E) NPC2 proteins were resolved by SDS-PAGE under nonreducing (NR) and reducing (R) conditions and visualized by Coomassie Brilliant Blue staining. [file Image_1.tif]

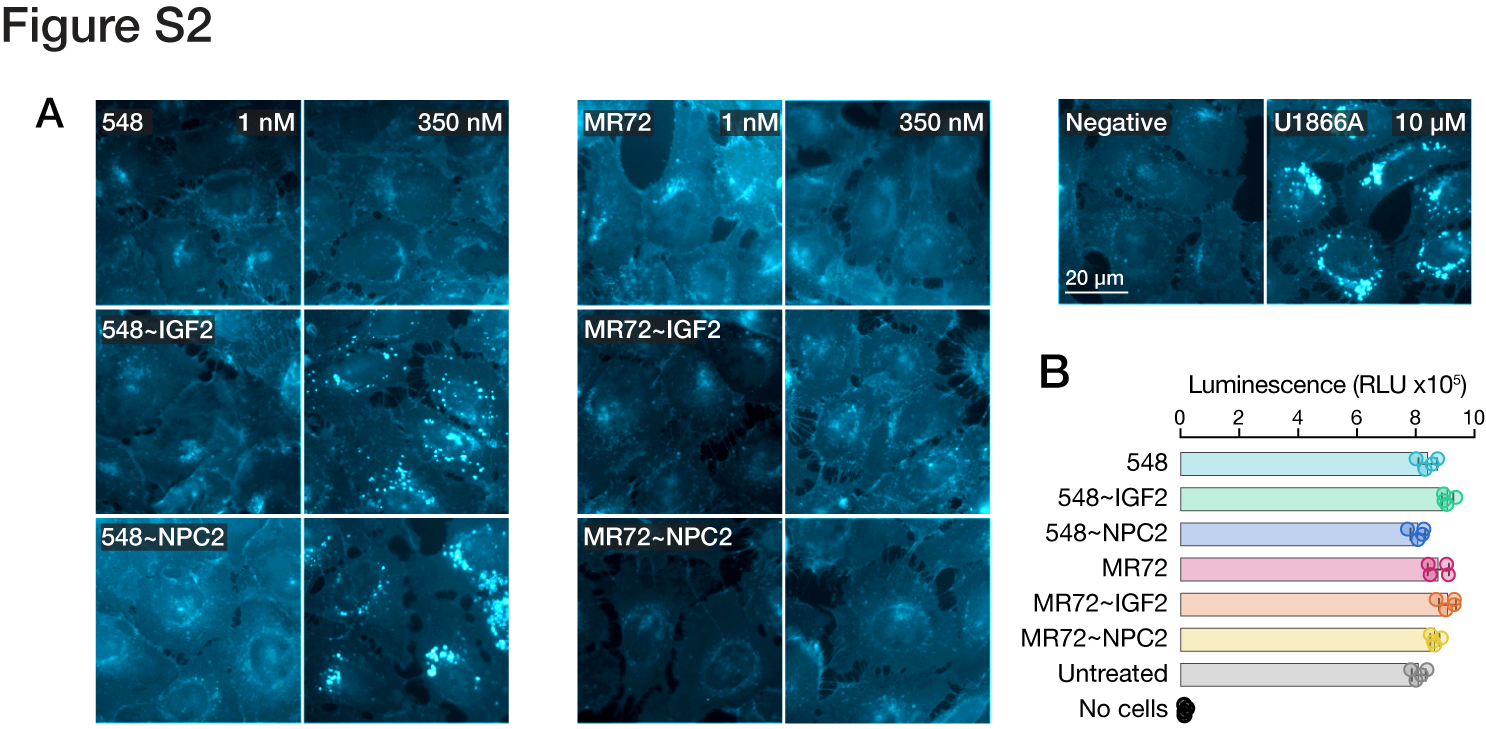

Supplement: Supplementary Figure S2 — mAb-548–containing bsAbs cause endo/lysosomal free cholesterol accumulation but do not affect cell viability. (A) Filipin staining of U2OS cells treated with the indicated antibodies (1 nM or 350 nM) or U18666A (positive control (54); 10 µM) for 14 h. (B) U2OS cell viability was determined by CellTiter-Glo assay following incubation with the indicated antibodies (1 nM or 350 nM) for 14 h. Means ± SD are shown for 4 replicates from 2 independent experiments. [file Image_2.tif]

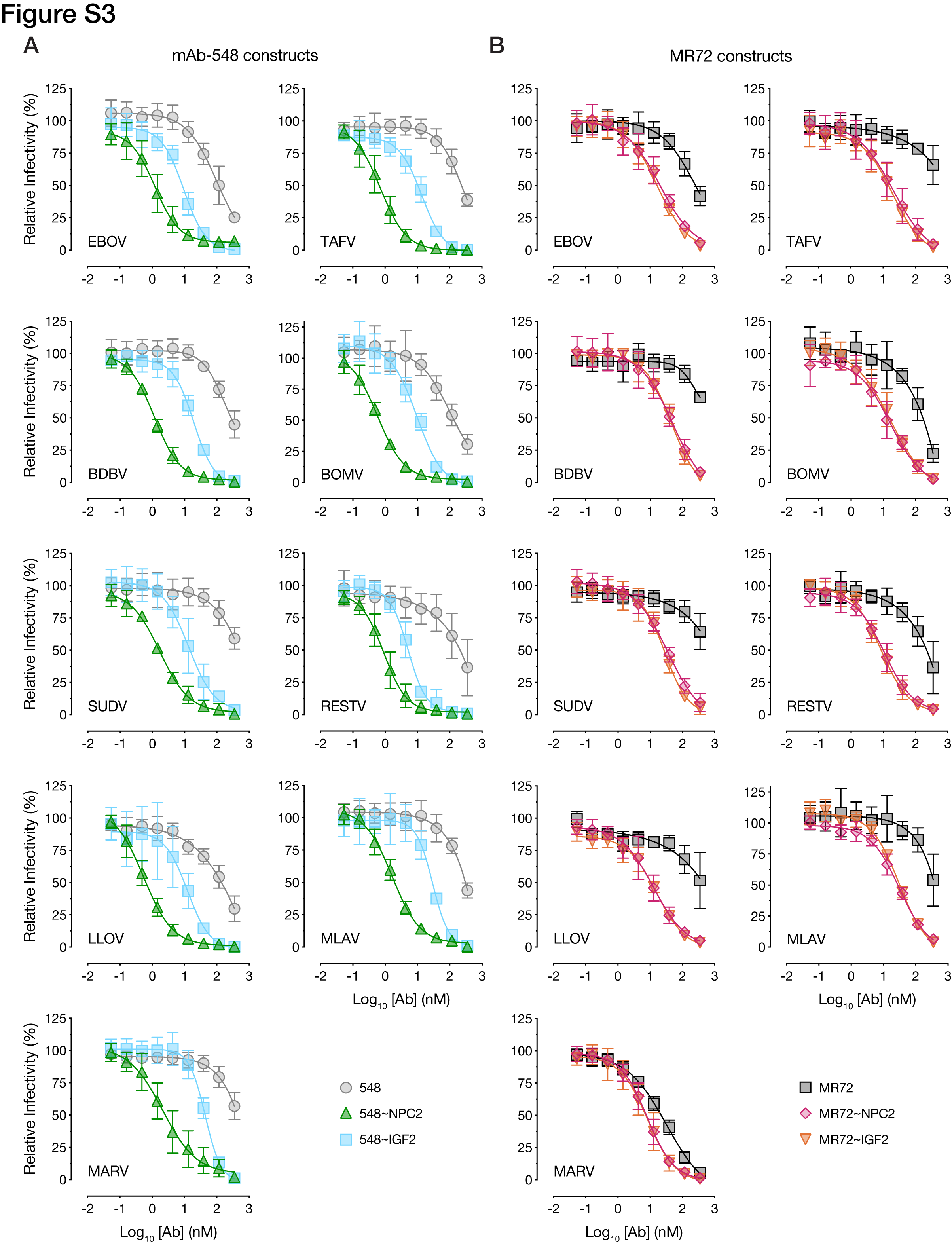

Supplement: Supplementary Figure S3 — Breadth of neutralization by Trojan horse bsAbs. Neutralization curves for (A) the mab-548 Trojan horse bsAb panel and (B) the MR72 Trojan horse bsAb panel against rVSVs bearing filovirus glycoproteins. IC50 values were calculated from curves for Figure 8A . Means ± SD are shown for 4–6 replicates from 2–3 independent experiments. EBOV, Ebola virus; TAFV, Tai Forest virus; BDBV, Bundibugyo virus; BOMV, Bombali virus; SUDV, Sudan virus; RESTV, Reston virus; LLOV, Lloviu virus; MLAV, Mengla virus; MARV, Marburg virus. [file Image_3.tif]

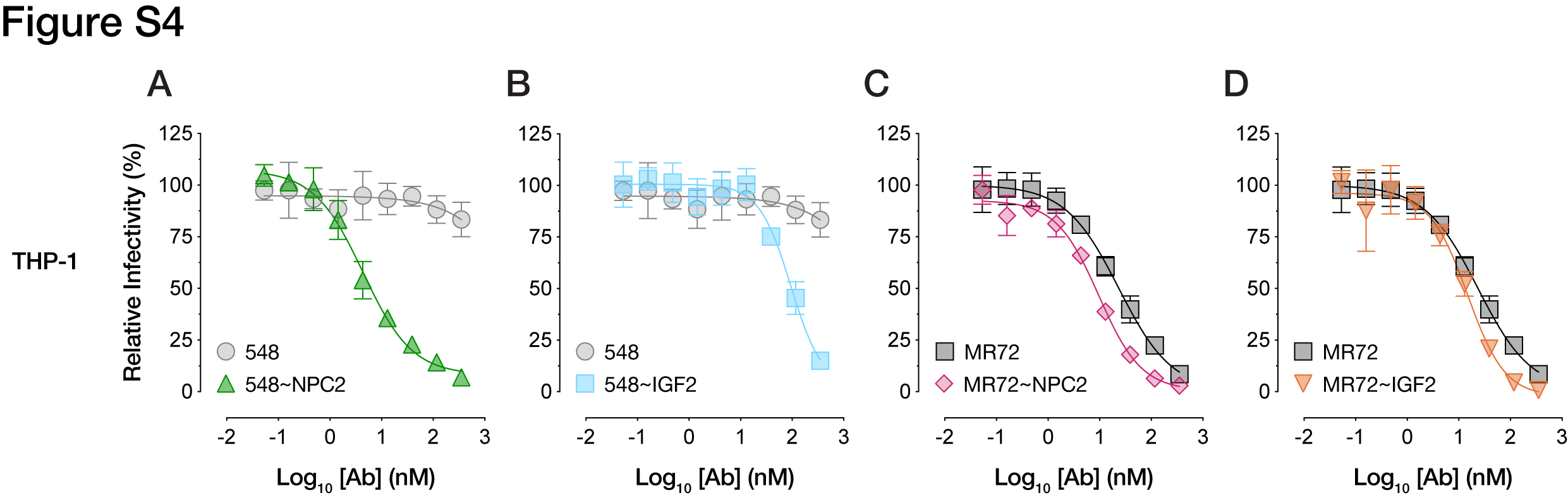

Supplement: Supplementary Figure S4 — NPC2- and IGF2-tagged bsAbs neutralize VSV-MARV in THP-1 cells. Neutralization activity of (A-B) mAb-548–containing bsAbs and (C-D) MR72-containing bsAbs against rVSV-MARV GP in differentiated THP-1 cells. Infection was measured by automated counting of eGFP+ cells and normalized to infection in absence of antibody. Means ± SD are shown for 4 replicates from 2 independent experiments. [file Image_4.tif]
